# Interobserver Variability and Comparison of Liquid-Based Cytology Versus Conventional Pap Smear for Cervical Cancer Screening in a High-Risk Population

**DOI:** 10.7759/cureus.87210

**Published:** 2025-07-03

**Authors:** Mariana S Chartian, Gautam Chellani, Meeta Singh, Nimisha Dhankar, Gurmeet Singh, Garima Rakheja, Rachita Garg, Gauri Gandhi, Nita Khurana, Shramana Mandal

**Affiliations:** 1 Pathology, Maulana Azad Medical College, New Delhi, IND; 2 Medicine, Maulana Azad Medical College, New Delhi, IND; 3 Epidemiology and Public Health, Postgraduate Institute of Medical Education and Research, Chandigarh, IND; 4 Community Medicine, Maulana Azad Medical College, New Delhi, IND; 5 Pathology and Laboratory Medicine, Dr. Ram Manohar Lohia Hospital, New Delhi, IND; 6 Obstetrics and Gynecology, Maulana Azad Medical College, New Delhi, IND

**Keywords:** cervical cancer, cervical cytology, conventional pap smear, interobserver variability, liquid-based cytology

## Abstract

Background and aim

The conventional Pap smear (CPS), while long considered the most effective cervical cancer (CC) screening tool in India, has increasingly come under scrutiny for its validity and reproducibility. Liquid-based cytology (LBC) is a relatively newer technique that offers a cleaner background and fewer unsatisfactory smears. This study aimed to assess interobserver variability and compare the diagnostic accuracy of LBC and CPS in the CC screening of high-risk patients.

Materials and methods

Split smears for CPS and LBC were prepared from 402 high-risk patients. CPS slides were stained using the Papanicolaou method. All CPS and LBC slides were initially reviewed by one cytopathologist and subsequently reevaluated by a second cytopathologist, with reporting based on the 2014 Bethesda System. In cases of discrepancy, the final diagnosis was determined by a third, senior cytopathologist. Biopsy confirmation was available in 52 cases. Interobserver variation was assessed using Kappa (κ) statistics, while intraobserver variation (between CPS and LBC) was evaluated using Pearson’s correlation coefficient (r).

Results

The strength of interobserver agreement was classified as very good for LBC (κ = 0.95) and good for CPS (κ = 0.79). Intraobserver agreement was high for observer 1 (r = 0.51) and moderate for observer 2 (r = 0.48). Diagnostic accuracy was 100% for LBC and 70% for CPS. CPS had significantly more unsatisfactory smears compared to LBC (p < 0.05).

Conclusions

Interobserver variability plays a critical role in diagnostic accuracy, patient care, and prognosis and has important medicolegal implications. In this study, interobserver variability was found to be higher in CPS than in LBC, highlighting LBC’s greater reliability in CC screening among high-risk patients.

## Introduction

According to Globocan 2020 statistics, cervical cancer (CC) is the second most common cancer affecting women in India [1]. Compared to developed nations, India bears a significantly higher burden of CC-related mortality, accounting for over 25% of global CC deaths and 33% of cases in Asia [2,3]. These alarming figures underscore the urgent need for effective intervention. The need is even more critical among women at high risk (WHR), who are more susceptible to long-term complications and rapid disease progression.

CC is one of the few malignancies that can be detected early, as it advances through identifiable precancerous stages known as cervical intraepithelial neoplasia (CIN). Therefore, implementing a reliable screening method is essential for enabling timely intervention and addressing this pressing public health issue [4]. In India, commonly used screening techniques include visual inspection with acetic acid, as well as cytological methods such as the conventional Pap smear (CPS) and liquid-based cytology (LBC) [5]. LBC is a relatively newer technique compared to CPS and offers several advantages, including a lower rate of unsatisfactory samples, quicker turnaround time, and a cleaner background that allows better visualization of cellular morphology [6]. Although various studies have evaluated the efficacy of CPS versus LBC, data focusing specifically on high-risk women remain limited, an important gap this study seeks to address [7].

Since CPS and LBC slides are typically evaluated by a single pathologist, the diagnostic outcome relies heavily on individual interpretation. As a result, any misdiagnosis can have serious consequences for patient care [7]. This highlights the importance of assessing interobserver variability to determine the consistency and reliability of cytological interpretations. The present study aimed to evaluate interobserver variability and compare the diagnostic accuracy of LBC and CPS in CC screening among high-risk patients.

## Materials and methods

Participants

This prospective comparative study was conducted over a period of 21 months (December 2019 to August 2022) and included a total of 402 female participants. The participants were women attending the obstetrics and gynecology outpatient department of a tertiary care teaching hospital. Inclusion criteria comprised patients classified as high-risk, based on parameters used in a study conducted at King George Medical University [4]: (1) age ≥ 40 years; (2) symptomatic presentation (e.g., contact bleeding or postmenopausal bleeding); and (3) high parity (three or more childbirths). Pregnant women and known cases of CIN or CC were excluded from the study.

Study design

Twin samples for both CPS and LBC were collected from high-risk patients by the Department of Obstetrics and Gynecology. Where clinically indicated, colposcopy-guided biopsy (CGB) was also performed. All samples (CPS, LBC, and CGB) were sent to the Department of Pathology, appropriately labeled, and examined according to standard protocols. The cytology results (CPS and LBC) were correlated with histopathology findings from CGB wherever available.

Methodology

For each participant, split-sample Pap smears were collected in accordance with standard operating procedures after obtaining informed consent (signature or thumb impression) following an explanation of the study. Relevant clinical history and examination findings were documented on a case study form, along with contact details for possible follow-up. CGB was performed in cases where abnormalities were observed on CPS or LBC. However, due to poor follow-up compliance, CGB reports were only available for 52 out of the 402 participants.

All Pap smear samples (CPS and LBC) were examined independently by two cytopathologists of equal experience to assess interobserver variability. Both pathologists were blinded to each other’s findings and to the results of all three modalities (CPS, LBC, and CGB, where applicable). In cases of disagreement, a third senior cytopathologist provided the final diagnosis. All cytological findings were classified using the 2014 Bethesda System (TBS 2014) for reporting cervical cytology. A cytological diagnosis was considered positive if findings were atypical squamous cells of undetermined significance (ASC-US) or higher. On histopathological examination, CIN I or above was considered a positive result.

Data entry and statistical analysis

Data were compiled and entered into a Microsoft Excel spreadsheet (Microsoft Corporation, Redmond, WA, USA). Patient names were anonymized using alphanumeric codes to ensure confidentiality. Statistical analysis was performed using IBM SPSS Statistics for Windows, Version 26.0 (Released 2018; IBM Corp., Armonk, NY, USA). The chi-square test was used to compare the number of unsatisfactory samples and to evaluate the association between cytological findings in CPS and LBC. A p-value < 0.05 was considered statistically significant. Interobserver agreement was calculated using Cohen’s Kappa (κ) statistics, and intraobserver agreement was assessed using Pearson’s correlation coefficient (r).

Ethical considerations

The study received ethical approval from the institutional ethics committee (approval number F.1/IEC/MAMC/90/02/2022/No 103). A patient information sheet outlining the study’s objectives, methodology, and other relevant details was provided to all potential participants. Informed written consent was obtained prior to their inclusion in the study.

## Results

Participant characteristics

Gynecological Pap smears were prospectively collected from 402 high-risk women, aged between 27 and 77 years, with a mean age of 47.8 ± 8.8 years. Parity among participants ranged from 0 to 9 childbirths, with a mean parity of 3.63 ± 0.89. These 402 participants were selected from a total of 1,353 twin samples of CPS and LBC received during the study period, following the application of the exclusion criteria.

Presenting features

The most common presenting complaint among participants was lower abdominal pain (58%), followed by irregular menstruation (20%). Other reported symptoms included white vaginal discharge, postcoital bleeding, postmenopausal bleeding, burning micturition, and the sensation of a mass descending per vaginum.

Comparison of CPS and LBC smears for both observers

The number of unsatisfactory smears was significantly higher with CPS than with LBC, as recorded by both observers. Observer 1 reported 129 unsatisfactory CPS smears, while observer 2 recorded 111; in contrast, both observers identified only 42 unsatisfactory smears with LBC. Intraobserver agreement, referring to the consistency in diagnosis between CPS and LBC for the same case, was found to be moderate for both observers: 0.51 for observer 1 and 0.48 for observer 2. Interobserver agreement was calculated using Cohen’s κ statistic and showed an almost perfect level of agreement for LBC (κ = 0.95) and a substantial level of agreement for CPS (κ = 0.79) (Table 1).

**Table 1:** Reports from two observers on LBC and CPS AGC-FN, atypical glandular cells, favor neoplastic; AGC-NOS, atypical glandular cells, not otherwise specified; ASC-H, atypical squamous cells, cannot exclude high-grade squamous intraepithelial lesion; ASC-US, atypical squamous cells of undetermined significance; BCC, benign cellular changes; BV, bacterial vaginosis; CPS, conventional Pap smear; HSIL, high-grade squamous intraepithelial lesion; LBC, liquid-based cytology; LSIL, low-grade squamous intraepithelial lesion; NILM, negative for intraepithelial lesion or malignancy; SCC, squamous cell carcinoma

Diagnosis category	CPS: Observer 1	CPS: Observer 2	LBC: Observer 1	LBC: Observer 2
Unsatisfactory	129	111	42	42
NILM	203	194	196	194
NILM-BV	13	23	46	46
NILM-*Trichomonas vaginalis*	1	1	1	1
NILM-*Candida*	3	5	16	16
NILM-BCC	12	15	16	16
NILM-atrophy	6	14	14	16
NILM-radiotherapy	0	0	3	3
NILM-inflammation	2	5	9	9
ASC-US	22	23	30	31
ASC-H	4	6	6	7
LSIL	3	1	6	5
HSIL	0	1	10	9
AGC-NOS	0	0	4	4
AGC-FN	0	0	1	1
SCC	4	3	2	2
Total	402	402	402	402

Epithelial abnormalities were more effectively detected using LBC than CPS (p < 0.05). A total of 59 samples were identified with abnormal morphology on LBC by both observers, compared to 33 and 34 samples on CPS by observers 1 and 2, respectively (Table 2).

**Table 2:** Positive cytological diagnoses on the split-sample smears AGC-FN, atypical glandular cells, favor neoplastic; AGC-NOS, atypical glandular cells, not otherwise specified; ASC-H, atypical squamous cells, cannot exclude high-grade squamous intraepithelial lesion; ASC-US, atypical squamous cells of undetermined significance; CPS, conventional Pap smear; HSIL, high-grade squamous intraepithelial lesion; LBC, liquid-based cytology; LSIL, low-grade squamous intraepithelial lesion; SCC, squamous cell carcinoma

Diagnosis category	CPS: Observer 1	LBC: Observer 1	CPS: Observer 2	LBC: Observer 2
ASC-US	22	30	23	31
ASC-H	4	6	6	7
LSIL	3	6	1	5
HSIL	0	10	1	9
AGC-NOS	0	4	0	4
AGC-FN	0	1	0	1
SCC	4	2	3	2
Total	33	59	34	59

Discordance in the diagnosis of epithelial abnormalities between the two cytological methods

When the findings of observers 1 and 2 were verified by a third observer or biopsy (when available), LBC was found to be more accurate than CPS in detecting epithelial cell abnormalities (Table 3).

**Table 3:** Discordance in the diagnosis of epithelial abnormalities between the two cytological methods AGC-FN, atypical glandular cells, favor neoplastic; AGC-NOS, atypical glandular cells, not otherwise specified; ASC-H, atypical squamous cells, cannot exclude high-grade squamous intraepithelial lesion; ASC-US, atypical squamous cells of undetermined significance; BCC, benign cellular changes; CIN, cervical intraepithelial neoplasia; CPS, conventional Pap smear; HSIL, high-grade squamous intraepithelial lesion; LBC, liquid-based cytology; LSIL, low-grade squamous intraepithelial lesion; NILM, negative for intraepithelial lesion or malignancy; SCC, squamous cell carcinoma

Case	CPS: Observer 1	CPS: Observer 2	Observer 3/biopsy	LBC: Observer 1	LBC: Observer 2	Observer 3/biopsy
1	ASC-US	Unsatisfactory	ASC-US	ASC-US	NILM-BCC	CIN I
2	NILM	ASC-US	CIN I	ASC-US	NILM	ASC-US
3	NILM-BCC	ASC-US	Chronic cervicitis	NILM-BCC	ASC-US	Chronic cervicitis
4	ASC-US	NILM	Normal	NILM	ASC-US	CIN I
5	ASC-US	Unsatisfactory	Unsatisfactory	LSIL	ASC-US	CIN I
6	ASC-US	ASC-H	Chronic cervicitis	HSIL	ASC-H	CIN III
7	ASC-US	ASC-H	CIN III	LSIL	ASC-US	LSIL
8	NILM	ASC-US	Normal	HSIL	ASC-H	SCC cervix
9	LSIL	ASC-US	Chronic cervicitis	AGC-NOS	AGC-FN	Adenocarcinoma cervix
10	NILM-BCC	ASC-US	Normal	AGC-FN	AGC-NOS	AGC-NOS
11	LSIL	ASC-US	ASC-US	-	-	-
12	Unsatisfactory	ASC-US	Unsatisfactory	-	-	-
13	SCC	HSIL	Adenocarcinoma cervix	-	-	-
Total discordant pairs (CPS)	13			Total discordant pairs (LBC)		10

Histopathology reports were available for 52 cases in which abnormalities were detected on either CPS or LBC (Table 4).

**Table 4:** Correlation of histopathology findings with CPS and LBC reports AGC-FN, atypical glandular cells, favor neoplastic; AGC-NOS, atypical glandular cells, not otherwise specified; ASC-H, atypical squamous cells, cannot exclude high-grade squamous intraepithelial lesion; ASC-US, atypical squamous cells of undetermined significance; BCC, benign cellular changes; BV, bacterial vaginosis; CIN, cervical intraepithelial neoplasia; CPS, conventional Pap smear; HSIL, high-grade squamous intraepithelial lesion; LBC, liquid-based cytology; LSIL, low-grade squamous intraepithelial lesion; NILM, negative for intraepithelial lesion or malignancy; SCC, squamous cell carcinoma

Case	CPS diagnosis	LBC diagnosis	Histopathology report
1	ASC-US	HSIL	Endometrial carcinoma
2	SCC	ASC-H	Adenocarcinoma cervix
3	ASC-US	ASC-H	Chronic cervicitis
4	ASC-US	HSIL	Squamous cell carcinoma cervix
5	ASC-US	LSIL	Chronic cervicitis
6	NILM	ASC-US	CIN I
7	ASC-US	LSIL	Chronic cervicitis
8	ASC-US	HSIL	CIN III
9	ASC-US	NILM	Normal
10	ASC-H	NILM	Normal
11	ASC-US	NILM	Normal
12	ASC-US	NILM-atrophic smear	Normal
13	Unsatisfactory	ASC-US	Normal
14	ASC-H	NILM	Normal
15	ASC-US	LSIL	Chronic cervicitis
16	ASC-US	NILM	Normal
17	ASC-US	NILM-BCC	Inflammation
18	LSIL	ASC-US	Chronic cervicitis
19	NILM	ASC-US	Normal
20	ASC-US	NILM-BV	Normal
21	ASC-US	NILM-BV	Normal
22	ASC-H	NILM-BV	Normal
23	ASC-US	NILM	Normal
24	ASC-H	NILM	Normal
25	NILM	ASC-US	Chronic cervicitis
26	ASC-H	NILM	Normal
27	NILM	ASC-H	Chronic cervicitis
28	Unsatisfactory	ASC-US	Normal
29	NILM	ASC-H	CIN I
30	NILM	ASC-US	Chronic cervicitis
31	NILM	ASC-US	CIN I
32	NILM	ASC-H	CIN I
33	ASC-US	HSIL	CIN III
34	ASC-US	NILM-BV	Chronic cervicitis
35	Unsatisfactory	HSIL	Squamous cell carcinoma cervix
36	ASC-US	ASC-H	CIN III
37	NILM	ASC-US	CIN II
38	NILM-BV	ASC-US	Chronic cervicitis
39	ASC-US	AGC-NOS	Normal
40	NILM	ASC-US	Inflammation
41	ASC-H	NILM-BCC	Chronic cervicitis
42	NILM	ASC-US	Chronic cervicitis
43	ASC-US	HSIL	Endometrial carcinoma
44	ASC-H	ASC-US	Chronic cervicitis
45	SCC	ASC-H	Adenocarcinoma cervix
46	ASC-H	ASC-US	CIN I
47	ASC-US	LSIL	CIN I
48	Unsatisfactory	ASC-US	Chronic cervicitis
49	ASC-US	HSIL	Squamous cell carcinoma cervix
50	ASC-US	NILM	Normal
51	ASC-US	HSIL	CIN II
52	Unsatisfactory	AGC-FN	Adenocarcinoma cervix

## Discussion

CPS has long been the primary modality for CC screening in India [8]. However, due to its higher rate of unsatisfactory smears and the presence of a less clear background, LBC is now regarded as a superior screening method for CC. Nevertheless, because LBC is more expensive, CPS continues to be the preferred tool in low-resource regions of India [9].

In the present study, LBC yielded a lower percentage of unsatisfactory smears compared to CPS (10.44% for LBC as reported by both observers, versus 32.08% and 27.61% for CPS by observers 1 and 2, respectively). Although the rate of unsatisfactory LBC smears in this study was higher than reported in previous literature [10,11], this may be attributed to the inclusion of the period during which the equipment was being installed and standardized, leading to technical issues that affected sample processing and may have confounded the data. Low cellularity was identified as a major cause of unsatisfactory LBC samples in this study, a finding consistent with the observations of Singh et al. [11]. According to Pankaj et al. [9] and Siebers et al. [12], the cleaner background in LBC facilitates better visualization of cells, making low cellularity the primary reason for unsatisfactory LBC smears. In contrast, for CPS, unsatisfactory smears were commonly due to both low cellularity and obscuring factors such as blood or inflammatory cells. Since CPS involves direct deposition of biological material onto the slide, unlike the treated preparation in LBC, the presence of additional components such as red blood cells and inflammatory cells alongside cervical cells from the transformation zone is expected. Similar findings have been reported in other studies [8,13].

In our study, *Gardnerella vaginalis *(indicative of a shift in vaginal flora) was the most commonly identified pathogenic organism, detected in 46 LBC slides by both observers and in 13 and 23 CPS slides by observers 1 and 2, respectively. *Trichomonas vaginalis *and *Candida *species were also detected. In total, 63 LBC slides were found to harbor pathogenic organisms according to both observers, compared to 17 and 29 CPS slides for observers 1 and 2, respectively. These findings are consistent with the results of Sherwani et al. [14], who reported better detection of pathogenic organisms with LBC, but contrast with the findings of Sharma et al. [8]. This discrepancy may be explained by the higher proportion of unsatisfactory CPS samples in both our study and Sherwani et al. [14].

Regarding epithelial abnormalities, LBC proved more effective in detecting ASC-US compared to CPS, aligning with the findings of Pankaj et al. [9] and Ilter et al. [15]. However, Sharma et al. [8] and Ezzat and Abusinna [10] reported opposite results. Ezzat and Abusinna suggested that some cells may appear normal on LBC but abnormal on CPS. In our study, the higher number of unsatisfactory CPS samples likely contributed to the lower ASC-US detection rate, helping to explain this variation. Additionally, LBC showed a significantly higher number of AGC-NOS and HSIL diagnoses compared to CPS. When correlated with biopsy findings, LBC demonstrated greater accuracy in identifying epithelial abnormalities. These findings are in agreement with a multicenter, US-based study [16] but contrast with a study conducted in Bihar, India [17]. In our study, biopsy was only performed in cases where epithelial abnormalities were detected, so only a subset of cases had biopsy data available for comparison.

The interobserver agreement was found to be 0.95 (indicating almost perfect agreement) for LBC and 0.79 (indicating substantial agreement) for CPS. Most disagreements with CPS were related to the diagnosis of negative for intraepithelial lesion or malignancy, negative for intraepithelial lesion or malignancy-bacterial vaginosis, negative for intraepithelial lesion or malignancy-benign cellular changes, and determinations of sample adequacy. A similar split-sample study conducted in Thailand using 777 samples also reported notable differences in interobserver agreement between LBC and CPS, although their major disagreements involved ASC-US and ASC-H diagnoses [18]. Our findings differ from those of Chhieng et al. [19], who reported similar interobserver agreement for both LBC and CPS, but their study was limited to only 20 split samples, which may account for the discrepancy. The interobserver agreement for CPS in our study was comparable to some studies [19,20] and higher than in others [21].

The intraobserver agreement (concordance) between LBC and CPS results was 0.51 (high) for observer 1 and 0.48 (moderate) for observer 2. These values contrast with those reported in a 2019 study from Uttar Pradesh [22], which found a concordance of 98%, and with a study from Egypt that reported an agreement of 0.736 between LBC and CPS [10]. The key difference likely stems from the higher rate of unsatisfactory CPS samples in our study. When unsatisfactory samples are excluded, the concordance in our study is comparable to that in previous studies.

This study included only WHR for CC, making it one of the first studies to focus exclusively on this population. Further research is needed to compare the risk of CIN and CC in WHR populations versus the general population. Such data would enhance screening protocols and facilitate early detection and treatment of CIN and CC, leading to better outcomes. Interobserver variability holds particular significance in WHR populations, as misdiagnoses can delay treatment, resulting in poorer outcomes due to the higher likelihood of dysplastic changes. A major strength of the present study is its large sample size, specifically among high-risk women.

## Conclusions

LBC surpasses CPS in enhancing the effectiveness of CC screening by increasing the detection rates of neoplastic and preneoplastic lesions while minimizing the overdiagnosis of benign conditions. Even when strict adherence to TBS guidelines is maintained, perfect inter- and intraobserver reproducibility in reporting cervical smears is not guaranteed. To ensure accurate reporting, discrepancies among observers should be discussed and resolved before the final report is issued to the treating gynecologist. Accurate diagnosis is particularly crucial for WHR of developing CC, as even minor interpretive errors may lead to missed opportunities for early intervention or result in unnecessary anxiety and procedures. Timely and precise identification of preneoplastic lesions can significantly improve outcomes by enabling early treatment and reducing the risk of progression to invasive cancer. Therefore, successful CC prevention, especially in high-risk populations, depends on consistency, standardization, and consensus in cytological reporting.
